# The Barthel Index in Predicting Response to Cardiac Resynchronization Therapy and Clinical Outcomes in Patients With Heart Failure

**DOI:** 10.1002/clc.70389

**Published:** 2026-07-09

**Authors:** Kailun Zhu, Haojie Zhu, Xiaofei Li, Jiawei Chen, Dingkun Lu, Xiaohan Fan

**Affiliations:** ^1^ Cardiac Arrhythmia Center, Fuwai Hospital, National Center for Cardiovascular Diseases, Chinese Academy of Medical Sciences and Peking Union Medical College Beijing China

**Keywords:** barthel index, cardiac resynchronization therapy, echocardiographic response, heart failure, left bundle branch area pacing

## Abstract

**Background:**

Cardiac resynchronization therapy (CRT) response in heart failure (HF) patients is multifactorial, with limited data on its association with activities of daily living assessed by the Barthel index (BI).

**Objective:**

This prospective observational study investigated the predictive value of BI for CRT response and long‐term clinical outcomes in non‐ischemic HF patients with left bundle branch block (LBBB).

**Methods:**

A total of 271 consecutive non‐ischemic HF patients undergoing CRT with left ventricular ejection fraction (LVEF) ≤ 35% and LBBB were enrolled. BI was evaluated pre‐implantation. The primary endpoint was CRT response (absolute LVEF improvement ≥ 5% at 6 months). The secondary composite endpoint included all‐cause mortality and HF hospitalization (HFH).

**Results:**

Among 249 patients completing 6‐month follow‐up, the CRT response rate was 68.67%. Multivariate logistic regression identified BI (per 5‐point increment; OR = 1.37, 95% CI:1.12−1.67, *p* = 0.002) as an independent predictor of CRT response, with an optimal cutoff of 95. Combining BI with left ventricular end‐diastolic diameter and left bundle branch area pacing achieved the highest predictive efficacy (AUC = 0.78). Over a median 33.94‐month follow‐up, BI > 95 was associated with an 88% reduced risk of the composite endpoint (adjusted HR = 0.116, *p* < 0.001), driven primarily by marked HFH reduction. A non‐significant trend toward lower all‐cause mortality was observed, accompanied by sustained LVEF improvement (29.81% ± 5.01% to 51.71% ± 11.30%).

**Conclusion:**

BI might be a simple, cost‐effective independent predictor for CRT response and improved LVEF and prognosis in non‐ischemic HF patients with LBBB.

## Graphical abstract

Combining Barthel index, left ventricular end‐diastolic diameter, and left bundle branch area pacing optimally predicted cardiac resynchronization therapy response (AUC 0.78). Long‐term follow‐up showed that a Barthel index score > 95 correlated with reduced all‐cause mortality and heart failure hospitalization (HR 0.204) and improved left ventricular ejection fraction.

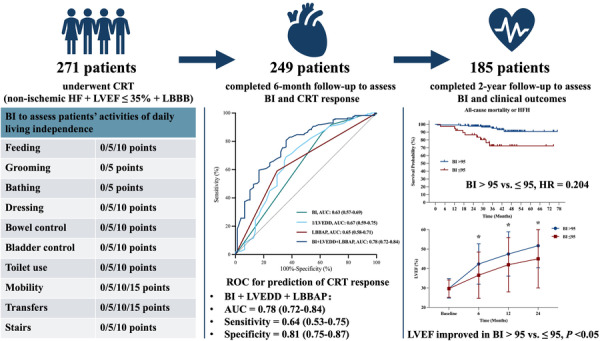

## 1 Introduction

As a classic cardiac resynchronization therapy (CRT) strategy, biventricular pacing (BiVP) effectively treats heart failure (HF) patients by improving ventricular synchronization to reduce all‐cause mortality and HF hospitalization (HFH) [1, 2]. However, 30%−40% of patients are non‐responsive to BiVP [3, 4]. Recent studies indicate that left bundle branch area pacing (LBBAP) derived CRT for HF patients achieves comparable or even superior efficacy to BiVP, but there are still some patients who do not respond [5, 6, 7]. Current predictors focus on cardiac structure, electrophysiology, or mechanical activity, which rely on high‐end and sophisticated equipment, thus hampering their widespread clinical application [8, 9, 10]. Predicting CRT response using economical, simple preoperative parameters remains a key challenge [11].

The Barthel index (BI), a core tool for activities of daily living assessment since 1965, quantifies functional independence via 10 categories of self‐care: feeding, moving from wheelchair to bed and returning, doing personal toilet, getting on and off the toilet, bathing self, walking on a level surface, ascending and descending stairs, dressing and undressing, continence of bowels, and controlling the bladder. Each score is evaluated using a scale of 0−15 points, with a perfect score equaling 100 points [12]. Characterized by simplicity (completable at the bedside in 5−10 min), low cost, and high reproducibility, BI comprehensively reflects cardiac function, skeletal muscle function, and systemic circulation in HF patients, factors that may influence CRT response. While the exact biological mechanisms remain to be elucidated, a high BI is hypothesized that indicating functional independence may be associated with less advanced myocardial remodeling and preserved exercise tolerance, which could potentially facilitate the recovery of ventricular synchronization after CRT. Conversely, a low BI indicating functional dependence may reflect more severe HF or significant comorbidities, which might attenuate the benefits of CRT. Currently, BI is seldom incorporated into CRT response prediction models. Thus, this study aims to explore the association between BI and CRT response in HF patients, and evaluate the predictive value of BI for the prognosis of HF patients received CRT.

## 2 Methods

### 2.1 Study Design and Population

**Figure 1:**
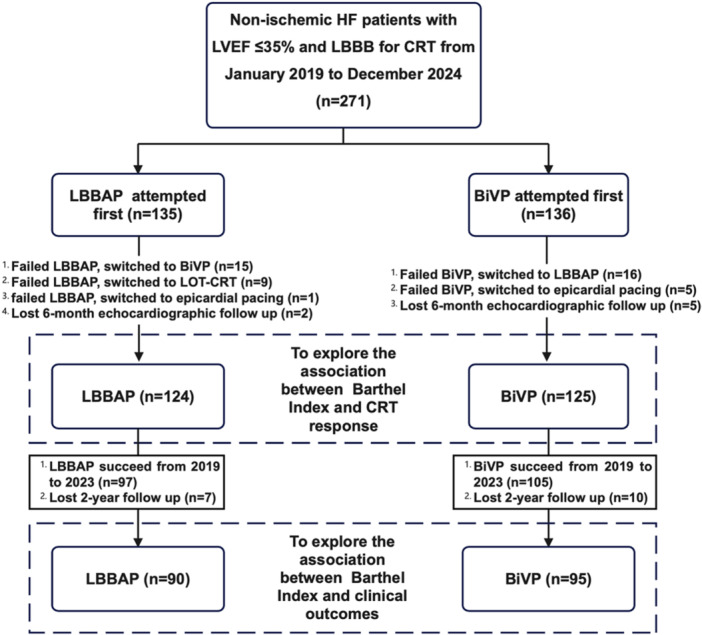
Flowchart of enrolled patients. Flowchart of enrolled patients in this study. BiVP, biventricular pacing; CRT, cardiac resynchronization therapy; HF, heart failure; LBBB, left bundle branch block; LBBAP, left bundle branch area pacing; LOT‐CRT, left bundle area pacing‐ optimized cardiac resynchronization therapy; LVEF, left ventricular ejection fraction.

This was a prospective, observational study at Fuwai Hospital, Chinese Academy of Medical Sciences, Peking Union Medical College. This prospective observational study enrolled consecutive patients between January 1, 2019, and December 31, 2024, at Fuwai Hospital. Follow‐up was completed on December 31, 2024. Patients enrolled between 2019 and 2023 were included in the long‐term outcome analysis, while those enrolled in 2023–2024 were excluded due to insufficient follow‐up duration. Consecutive non‐ischemic HF patients with a LVEF ≤ 35% and left bundle branch block (LBBB) who met CRT indications were enrolled. CRT strategies were determined by implanting physicians based on their clinical experience. Both BiVP and LBBAP were acceptable, and intraoperative crossover between these strategies was permitted to achieve optimal outcomes. All primary efficacy and outcome analyses were performed using an as‐treated approach, where patients were categorized strictly according to the final successful pacing modality they received, rather than the initially assigned strategy. However, patients who underwent LBBAP‐optimized CRT (LOT‐CRT) or received epicardial left ventricle (LV) lead implantation for BiVP were excluded. Other exclusion criteria included patients under 18 years of age, ischemic cardiomyopathy, non‐LBBB, refusal to provide written informed consent, and those with a life expectancy of less than 1 year. Figure 1 shows the flowchart of all enrolled patients. This study adhered to the Declaration of Helsinki and was approved by the Ethics Committee of Fuwai Hospital. Study data are available from the corresponding author on reasonable request (Table 1).

**Table 1:** Baseline characteristics of all enrolled patients.

Variables	Total (*n* = 271)
Age, year	59.80 ± 9.80
Male, *n* (%)	165 (60.89)
Height, cm	169.00 (160.00, 173.00)
Weight, kg	70.00 (60.00, 78.00)
SBP, mmHg	120.00 (110.00, 130.00)
DBP, mmHg	70.00 (64.00, 77.00)
Smoke, *n* (%)	91 (33.58)
Drink, *n* (%)	64 (23.62)
Hypertension, *n* (%)	118 (43.54)
Diabetes mellitus, *n* (%)	63 (23.25)
NYHA ≤ 2, *n* (%)	97 (35.79)
History of HF, year	3.00 (1.00, 8.00)
History of unexplained syncope or malignant VAs, *n* (%)	32 (11.81)
NT‐proBNP, ng/mL	890.00 (455.00, 2094.00)
Barthel index > 95, *n* (%)	210 (77.49)
Intrinsic QRS duration (ms)	178.59 ± 18.57
Echocardiography	
LVEDD, mm	67.00 (62.00, 75.00)
LVEF, %	29.58 ± 4.92
Medication	
Beta blockers	270 (99.63)
ACEI, ARB, ARNI	250 (92.25)
Spironolactone	262 (96.68)
SGLT2i	210 (77.49)
Diuretic	245 (90.41)

*Note:* Values are presented as mean ± SD or median (IQR) for continuous variables and numbers and percentages for categorical variables.

Abbreviations: ACEI, angiotensin‐converting enzyme inhibitor; ARB, angiotensin Ⅱ receptor blocker; ARNI, angiotensin receptor‐neprilysin inhibitor; DBP, diastolic blood pressure; HF, heart failure; LVEDD, left ventricular end‐diastolic diameter; LVEF, left ventricular ejection fraction; SBP, systolic blood pressure; SGLT2i, sodium‐glucose cotransporter 2 inhibitor; VAs, ventricular arrhythmias.

### 2.2 Implantation Procedures and Device Programming

LBBAP implantation was performed as previously described [13]. Briefly, the pacing lead was advanced into the interventricular septum from the right side to the left side. When the lead reached the deep left septum, a right bundle branch block (RBBB) morphology of the paced QRS was observed in lead V_1_. LBBAP was considered successful if deep septal lead placement was achieved with unipolar paced Qr or qR morphology in lead V_1_ along with any of the following: recording of left bundle branch (LBB) potential; demonstration of transition from nonselective to selective LBB/LV septal capture; LV activation time in lead V_5_ or V_6_ < 90 ms; programmed stimulation demonstrating differential capture; or lead V_6_−V_1_ interpeak interval > 44 ms [5, 14, 15]. After successful LBBAP implantation, devices were programmed to ensure exclusive LBBAP with atrioventricular delay optimization for the shortest paced QRS duration.

For BiVP, LV leads were implanted in a standard fashion targeting the basal posterolateral LV, using quadripolar leads whenever feasible [15]. The defibrillation lead was placed at the right ventricular septum or apex. Postoperative optimizations of atrioventricular and interventricular intervals were performed to achieve the shortest paced QRS duration. Patients with failed BiVP procedures were transferred to the LBBAP procedure. If both LBBAP and BiVP failed, epicardial LV lead implantation was performed for BiVP.

### 2.3 Clinical Data Collection and Follow‐up

Baseline patient demographics, current medications, and electrocardiographic and echocardiographic parameters were collected. Pacing parameters, including capture threshold, R‐wave amplitudes, impedance, and pacing percentage, were obtained at implantation and during device clinic for 3 months, 6 months, and then at regular intervals of 12 months. The optimal HF medical therapy was individually determined according to the patient's symptoms, physical examination findings, and vital signs. Echocardiographic examination was performed during device clinic follow‐up. The left ventricular ejection fraction (LVEF) and left ventricular end‐diastolic diameter (LVEDD) were calculated by the Simpson biplane method.

The primary endpoint was defined as CRT response, specified as an absolute improvement in LVEF of ≥ 5% at 6 months post‐implantation. Secondary clinical endpoints included: (1) the composite outcome of all‐cause mortality and HFH [7]; (2) individual adverse events, namely all‐cause mortality, HFH, and malignant ventricular arrhythmias (VAs) [7, 16]; (3) changes in echocardiographic parameters.

### 2.4 Statistical Analysis

All statistical analyses were performed using SPSS version 25.0 (IBM Corp, Armonk, NY, USA) and GraphPad Prism 10.6.1 (GraphPad Software Inc., San Diego, CA, USA). Continuous variables were presented as mean ± standard deviation (SD) or median (interquartile range, IQR) based on normality assessed by the Shapiro‐Wilk test, with normally distributed data compared using independent samples *t*‐test (two groups) or analysis of variance (ANOVA, three or more groups) and non‐normally distributed data analyzed via the Kruskal−Wallis *H* test, while categorical variables were expressed as frequencies (percentages) and compared using the *χ* ^2^ test or Fisher's exact test as appropriate for expected cell frequencies.

CRT response was defined as an absolute improvement in LVEF ≥ 5% at 6 months post‐implantation (dichotomized as “response” or “non‐response”), and univariate and multivariate logistic regression models were used to explore the association between the BI, treated as a continuous variable, and CRT response. Variables of clinical significance or a univariate *p* < 0.10 were included in the multivariate model, which was adjusted for potential confounding factors. The odds ratios (OR) with 95% confidence intervals (CI) were calculated. The Hosmer−Lemeshow test was performed to assess the goodness‐of‐fit of the multivariate logistic regression model, where a two‐tailed *p* > 0.05 indicated adequate model fit. Receiver operating characteristic (ROC) curves were constructed to evaluate the predictive efficacy of BI for CRT response, with the area under the curve (AUC) and 95% CI used to quantify discriminative ability, and the optimal cutoff value of BI determined via the Youden index, along with corresponding sensitivity, specificity, positive predictive value (PPV), and negative predictive value (NPV) reported.

For clinical outcomes of the composite outcome of all‐cause mortality and HFH, and individual adverse events of all‐cause mortality, HFH, and malignant VAs, patients were stratified into two groups based on the optimal BI cutoff value derived from ROC analysis; Kaplan−Meier (KM) survival curves were plotted to depict cumulative event‐free survival probabilities across BI subgroups, with the log‐rank test used to compare survival differences, and Cox proportional hazards regression models were employed to estimate hazard ratios (HR) and 95% CI for clinical outcomes (adjusted for the same confounding factors as the logistic regression model). A two‐tailed *p* < 0.05 was considered statistically significant.

## 3 Results

A total of 271 patients were enrolled, with a mean age of 59.80 ± 9.80 years and 60.89% (*n* = 165) being male. Smoking and alcohol consumption histories were reported in 33.58% (*n* = 91) and 23.62% (*n* = 64) of patients, while hypertension (43.54%, *n* = 118) and diabetes mellitus (23.25%, *n* = 63) were common comorbidities. Median HF duration was 3.00 years (1.00−8.00), and 11.81% (*n* = 32) had unexplained syncope or malignant VAs; 77.49% (*n* = 210) had a BI > 95. Mean intrinsic QRS duration was 178.59 ± 18.57 ms. Echocardiography showed a mean LVEDD of 67.00 (62.00, 75.00) mm and LVEF of 29.58% ± 4.92%. Medication adherence was high: beta blockers (99.63%, *n* = 270), ACEI/ARB/ARNI (92.25%, *n* = 250), spironolactone (96.68%, *n* = 262), SGLT2 inhibitors (77.49%, *n* = 210), and diuretics (90.41%, *n* = 245).

Based on the clinical experience of implanting physicians, CRT strategies were assigned as follows: 135 patients first attempted LBBAP, and 136 first attempted BiVP. Among the LBBAP‐first patients, 15 patients switched to BiVP, 9 to LOT‐CRT, and 1 to epicardial pacing due to LBBAP failure; in the BiVP‐first patients, 16 patients switched to LBBAP and five to epicardial pacing due to BiVP failure. Two patients received LBBAP and five patients received BiVP who failed to complete 6‐month echocardiographic follow‐up were excluded. Based on the final successful pacing modality they received, a total of 249 patients were included in the final as‐treated analysis, of which 124 patients were included in the LBBAP cohort and 125 in the BiVP cohort were included to explore the association between the BI and CRT response (Figure 1).

### 3.1 Association Between BI and CRT Response

After 6 months of follow‐up, the echocardiographic response rate to CRT in the total population was 68.67% (171/249). Subsequently, univariate and multivariate logistic regression analyses were performed to identify predictors of CRT response, with variables having a *p* < 0.10 in the univariate analysis included in the multivariate model (Table 2). In univariate analysis, CRT response was significantly associated with longer HF duration (OR = 0.92, 95% CI 0.87−0.97, *p* = 0.004), higher BI (per 5‐point increment; OR = 1.36, 95% CI 1.13−1.63, *p* = 0.001), LVEDD (OR = 0.94, 95% CI 0.91−0.96, *p* < 0.001), and LBBAP versus BiVP (OR = 3.45, 95% CI 1.94−6.13, *p* < 0.001). Syncope or malignant VAs history (OR = 0.50, 95% CI 0.23−1.08, *p* = 0.08), alcohol consumption (*p* = 0.078), hypertension (*p* = 0.105), and intrinsic QRS duration (*p* = 0.096) approached significance and were included in multivariate analysis. After adjustment, higher BI (per 5‐point increment; OR = 1.37, 95%CI 1.12−1.67, *p* = 0.002), larger LVEDD (OR = 0.96, 95% CI 0.92−0.98, *p* = 0.005), and LBBAP versus BiVP (OR = 2.88, 95% CI 1.53−5.41, *p* = 0.001) remained independent predictors. The Hosmer‐Lemeshow test confirmed that the multivariate logistic regression model had a good fit (*χ* ^2^ = 5.002, df = 8, *p* = 0.757), validating the reliability of the observed associations between BI, LVEDD, LBBAP and CRT response.

**Table 2:** Predictors of CRT response by univariate and multivariate logistic regression analyses.

Variables	Univariate logistic regression		Multivariate logistic regression	
	OR (95% CI)	*p*	OR (95% CI)	*p*
Age, year	1.01 (0.98–1.04)	0.549		
Male	0.85 (0.49–1.48)	0.561		
Height, cm	0.98 (0.94–1.01)	0.206		
Weight, Kg	0.99 (0.97–1.01)	0.304		
SBP	1.00 (0.99–1.02)	0.844		
DBP	1.01 (0.98–1.04)	0.562		
Smoke	0.63 (0.36–1.10)	0.102		
Drink	0.58 (0.32–1.06)	0.078	0.94 (0.46–1.92)	0.871
Hypertension	1.35 (0.72–2.52)	0.346		
Diabetes mellitus	0.80 (0.39–1.61)	0.526		
NYHA ≤ 2	0.91 (0.52–1.59)	0.734		
History of HF, year	0.92 (0.87–0.97)	0.004	0.95 (0.90–1.01)	0.107
History of unexplained syncope or malignant VAs	0.50 (0.23–1.08)	0.080	0.59 (0.24–1.47)	0.259
Barthel index (per 5‐point increment)	1.36 (1.13–1.63)	0.001	1.37 (1.12–1.67)	0.002
NT–proBNP	0.78 (0.65–0.94)	0.560		
Intrinsic QRS duration, ms	0.99 (0.97–1.00)	0.096	1.00 (0.98–1.02)	0.953
Echocardiography				
LVEDD, mm	0.94 (0.91–0.96)	< 0.001	0.96 (0.92–0.98)	0.005
LVEF, %	1.04 (0.98–1.11)	0.161		
Medication				
Beta blockers	0.00 (0.00–Inf)	0.987		
ACEI, ARB, ARNI	1.21 (0.43–3.41)	0.715		
Spironolactone	1.33 (0.26–6.85)	0.731		
SGLT2i	1.58 (0.85–2.94)	0.146		
Diuretic	1.26 (0.53–3.00)	0.596		
LBBAP versus BiVP	3.45 (1.94–6.13)	< 0.001	2.88 (1.53–5.41)	0.001

Abbreviations: ACEI, angiotensin–converting enzyme inhibitor; ARB, angiotensin Ⅱ receptor blocker; ARNI, angiotensin receptor–neprilysin inhibitor; BiVP, biventricular pacing; CRT, cardiac resynchronization therapy; DBP, diastolic blood pressure; HF, heart failure; LBBAP, left bundle branch area pacing; LVEDD, left ventricular end–diastolic diameter; LVEF, left ventricular ejection fraction; OR, odds ratio; SBP, systolic blood pressure; SGLT2i, sodium–glucose cotransporter 2 inhibitor; VAs, ventricular arrhythmias.

**Figure 2:**
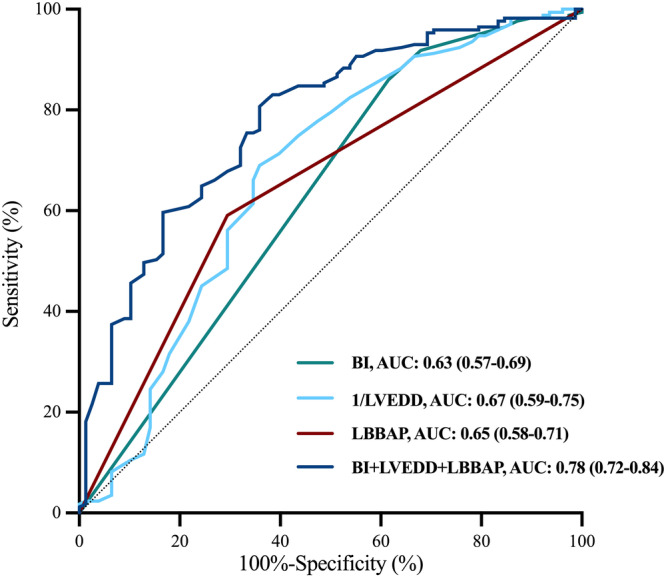
Receiver operating characteristic curves for prediction of cardiac resynchronization therapy response (CRT). Curves correspond to predictor BI (green, AUC = 0.63 [95% CI: 0.57−0.69]), 1/LVEDD (light blue, AUC = 0.67 [0.59−0.75]), LBBAP (dark red, AUC = 0.65 [0.58−0.71]), and their combination (dark blue, AUC = 0.78 [0.72−0.84]). BI, Barthel index; LBBAP, left bundle branch area pacing; LVEDD, left ventricular end‐diastolic diameter.

Table 3 and Figure 2 summarized the diagnostic performance of 1/LVEDD, BI, LBBAP, and their combination in predicting CRT response, with all metrics reported alongside 95% CI. For the continuous variable LVEDD, ROC curve analysis showed the AUC of 0.33 (95% CI: 0.25−0.40) for predicting CRT response, with the curve below the reference line (AUC = 0.5), indicating a negative association. After reciprocal transformation (1/LVEDD), repeat ROC analysis yielded an AUC of 0.67 (95% CI: 0.59−0.75), reflecting moderate predictive efficacy with sensitivity 0.56 (95% CI: 0.45−0.67) and specificity 0.75 (95% CI: 0.68−0.81) for predicted CRT response. Regarding the BI, ROC curve analysis demonstrated a comparable AUC of 0.63 (95% CI: 0.57−0.69). The optimal cutoff value derived from the Youden index was 97.5. However, given that BI scores are clinically recorded in 5‐ or 10‐point intervals, 95 was ultimately selected as the dichotomization threshold for practicality, with an AUC of 0.63 (95% CI: 0.57−0.69), specificity of 0.92 (95% CI 0.88−0.96). Consistent with this predictive performance, patients with BI > 95 exhibited a significantly higher CRT response rate compared to those with BI ≤ 95 (75.38% [147/195] vs. 44.44% [24/54], *p* < 0.001). Similarly, LBBAP alone yielded an AUC of 0.65 (95% CI: 0.58−0.71), a sensitivity of 0.71 (95% CI: 0.60−0.81), and a specificity of 0.59 (95% CI: 0.52−0.66).

**Table 3:** Diagnostic test evaluation indicators.

Variables	AUC (95% CI)	Accuracy (95% CI)	Sensitivity (95% CI)	Specificity (95%CI)	PPV (95% CI)	NPV (95% CI)	Cutoff
1/LVEDD	0.67 (0.59–0.75)	0.69 (0.63–0.75)	0.56 (0.45–0.67)	0.75 (0.68–0.81)	0.51 (0.40–0.61)	0.79 (0.73–0.85)	0.014
Barthel index	0.63 (0.57–0.69)	0.71 (0.65–0.77)	0.38 (0.28–0.49)	0.86 (0.81–0.91)	0.56 (0.42–0.69)	0.75 (0.69–0.81)	97.5
	0.63 (0.57–0.69)	0.73 (0.67–0.78)	0.32 (0.22–0.42)	0.92 (0.88–0.96)	0.64 (0.49–0.79)	0.75 (0.69–0.81)	95.0
LBBAP	0.65 (0.58–0.71)	0.63 (0.56–0.69)	0.71 (0.60–0.81)	0.59 (0.52–0.66)	0.44 (0.35–0.53)	0.81 (0.75–0.88)	/
Combination of LVEDD, Barthel index, and LBBAP	0.78 (0.72–0.84)	0.76 (0.70–0.81)	0.64 (0.53–0.75)	0.81 (0.75–0.87)	0.60 (0.50–0.71)	0.83 (0.77–0.89)	0.619

Abbreviations: AUC, area under the curve; LBBAP, left bundle branch area pacing; LVEDD, left ventricular end‐diastolic diameter; NPV, negative predictive value; PPV, positive predictive value.

Notably, the combination of LVEDD, BI, and LBBAP outperformed all individual predictors, achieving the highest predictive efficacy with an AUC of 0.78 (95% CI: 0.72−0.84), accuracy of 0.76 (95% CI: 0.70−0.81), sensitivity of 0.64 (95% CI: 0.53−0.75), specificity of 0.81 (95% CI: 0.75−0.87), PPV of 0.60 (95% CI: 0.50−0.71), and NPV of 0.83 (95% CI: 0.77−0.89). The addition of BI to the model containing only 1/LVEDD and LBBAP significantly improved the AUC from 0.71 to 0.78 (ΔAUC = 0.07, *p* = 0.023 by DeLong test), demonstrating a statistically significant incremental predictive value.

Two additional sensitivity analyses were performed using stricter CRT response definitions: for absolute LVEF increase ≥ 10% (Supporting Information S1: Table 3), BI remained an independent predictor (adjusted OR = 1.27 per 5‐point increment, 95% CI 1.15−1.38, *p* = 0.022); and for LVEF increase ≥ 15% (adjusted OR = 1.31 per 5‐point increment, 95% CI 1.14−1.60, *p* = 0.039) (Supporting Information S1: Table 4).

### 3.2 Long‐Term Clinical Outcomes of Patients Receiving CRT Stratified by BI Levels (> 95 vs. ≤ 95)

**Figure 3:**
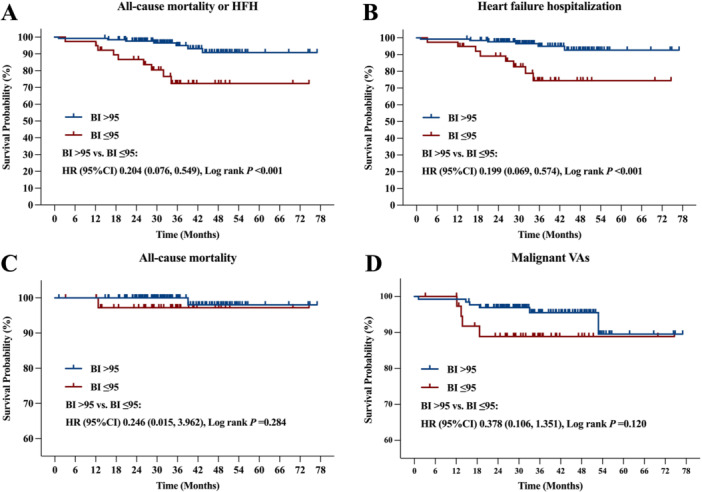
Kaplan−Meier curves for survival probability of clinical endpoints of patients receiving CRT stratified by BI levels (> 95 vs. ≤ 95). Kaplan−Meier curves for unadjusted survival probability of clinical endpoints of patients receiving cardiac resynchronization therapy response (CRT) stratified by Barthel index (BI) levels (> 95 vs. ≤ 95). (A) Kaplan−Meier curves for unadjusted risk of all‐cause mortality or heart failure hospitalization (HFH) of the BI > 95 group and the BI ≤ 95 group (HR = 0.204, 95% CI: 0.076−0.549, Log‐rank *p* < 0.001). (B) Kaplan−Meier curves for unadjusted risk of HFH of the BI > 95 group and the BI ≤ 95 group (HR = 0.199, 95% CI: 0.069−0.574, (Log‐rank *p* < 0.001). (C) Kaplan−Meier curves for unadjusted risk of all‐cause mortality of the BI > 95 group and the BI ≤ 95 group (HR = 0.246, 95%CI: 0.015‐3.962, Log‐rank *p* = 0.284). (D) Kaplan−Meier curves for unadjusted risk of malignant ventricular arrhythmias (VAs) of the BI > 95 group and the BI ≤ 95 group (HR = 0.378, 95%CI: 0.106‐1.351, Log‐rank *p* = 0.120). CI, confidence interval; HR, hazard ratio.

To investigate the association between the BI and long‐term CRT clinical outcomes, patients enrolled between 2019 and 2023 were included in this analysis, while those enrolled in 2023−2024 were excluded due to insufficient follow‐up duration. A total of 181 patients were enrolled from 249 individuals previously allocated to LBBAP (*n* = 124) and BiVP (*n* = 125) cohorts. These patients had successfully undergone CRT between 2019 and 2023, with a planned 2‐year follow‐up. No significant baseline characteristic disparities existed between this 2019−2023 subset (*n* = 181) and the 2023−2024 patient group (n = 68) (Supporting Information S1: Table 1). Thirteen patients were lost to 2‐year follow‐up, leaving 168 patients for clinical outcome survival analysis. Figure 3 presented KM survival curves for clinical outcomes in CRT patients, grouped by the BI cutoff value linked to CRT response: 95 was selected as the dichotomization threshold (stratifying patients into BI ≤ 95 and BI > 95 groups). Median follow‐up duration was 33.94 (26.67, 45.86) months, with a maximum follow‐up of about 78 months.

For the composite endpoint of all‐cause mortality and HFH (Figure 3A), the BI > 95 group maintained consistently high survival probability while the BI ≤ 95 group declined sharply (Log‐rank *p* < 0.001, HR = 0.204, 95% CI: 0.076−0.549); notably, this composite endpoint benefit was exclusively driven by a marked reduction in HFH risk, for the HFH endpoint alone (Figure 3B), the BI > 95 group had a 80.1% relative risk reduction versus BI ≤ 95 group (Log‐rank *p* < 0.001, HR = 0.199, 95% CI: 0.069−0.574). This protective association for all‐cause mortality and HFH remained statistically significant after adjusting for confounding factors; multivariate Cox proportional hazards regression revealed that the BI > 95 group had an adjusted HR of 0.116 (95% CI: 0.034−0.399, *p* < 0.001), corresponding to an approximate 88% relative risk reduction (Supporting Information S1: Table 2). In contrast, no significant difference was observed for the all‐cause mortality endpoint (Log‐rank *p* = 0.284, HR = 0.246, 95% CI: 0.015−3.962) (Figure 3C), although a trend toward lower mortality was noted in the BI > 95 group. The lack of statistical significance for mortality is likely due to the small number of death events in our cohort. For the malignant VAs endpoint (Figure 3D), the BI > 95 group had higher survival probability, though the difference only approached statistical significance (Log‐rank *p* = 0.120, HR = 0.378, 95% CI: 0.106−1.351). At baseline, 39 patients were in the BI ≤ 95 group and 129 in the BI > 95 group, with the number of at‐risk patients (without endpoint events) gradually decreasing in both groups as follow‐up progressed.

### 3.3 CRT Response and Long‐Term Clinical Outcomes of Patients Treated with LBBAP or BiVP Stratified by BI Levels (> 95 vs. ≤ 95)

**Figure 4:**
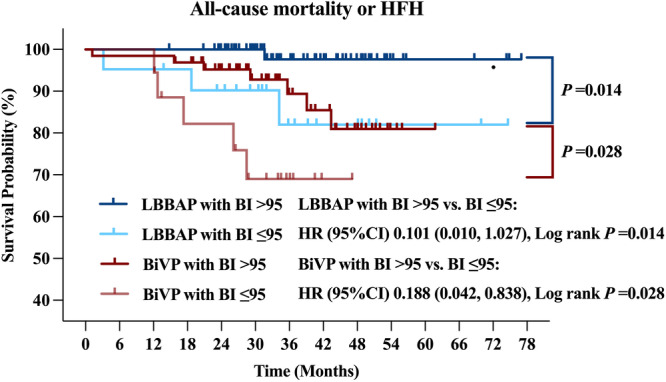
Kaplan−Meier curves for survival probability for all‐cause mortality or HFH among LBBAP and BiVP patients stratified by BI levels (> 95 vs. ≤ 95). Kaplan−Meier curves for survival probability for all‐cause mortality or heart failure hospitalization (HFH) among left bundle branch area pacing (LBBAP) and biventricular pacing (BiVP) patients stratified by Barthel index (BI) levels (> 95 vs. ≤ 95). Kaplan−Meier curves for unadjusted risk of all‐cause mortality and HFH for LBBAP with BI > 95 (dark blue) and BI ≤ 95 (light blue) (HR = 0.101, 95% CI: 0.010−1.027, Log‐rank *p* = 0.014). Kaplan−Meier curves for unadjusted risk of all‐cause mortality and HFH for BiVP with BI > 95 (dark red) and BI ≤ 95 (light red) (HR = 0.188, 95%CI: 0.042‐0.838, Log‐rank *p* = 0.028). CI, confidence interval; HR, hazard ratio.

Further subgroup analysis stratified by CRT strategy confirmed BI's persistent predictive value for the CRT response and clinical composite endpoint of all‐cause mortality or HFH. Comparison of clinical characteristics between patients treated with LBBAP or BiVP was showed in Supporting Information S1: Table 5. In both LBBAP and BiVP subgroups, BI remained an independent predictor of response (Supporting Information S1: Tables 6 and 7), with adjusted ORs of 1.57 (95% CI: 1.13−2.17) and 1.26 (95% CI: 1.09−1.42), respectively. KM survival curves for the four subgroups (LBBAP with BI > 95, LBBAP with BI ≤ 95, BiVP with BI > 95, BiVP with BI ≤ 95) revealed a gradual decline in endpoint‐free survival across all groups, with LBBAP subgroups consistently maintaining higher survival probabilities than their BiVP counterparts (Figure 4). Statistical analyses validated LBBAP combined with BI > 95 was associated with the most favorable long‐term outcomes (Log‐rank *p* = 0.014, HR = 0.101, 95% CI: 0.010−1.027). Similarly, among patients treated with BiVP, a BI > 95 was also linked to improved long‐term prognosis (Log‐rank *p* = 0.028, HR = 0.188, 95% CI: 0.042−0.838). These findings reinforcing the protective effect of BI > 95 observed in the full CRT cohort and highlighting strategy‐specific differences in BI's predictive utility for long‐term clinical outcomes of patients who received LBBAP or BiVP.

### 3.4 Long‐Term Echocardiographic Outcomes of Patients Receiving CRT Stratified by BI Levels (> 95 vs. ≤ 95)

**Figure 5:**
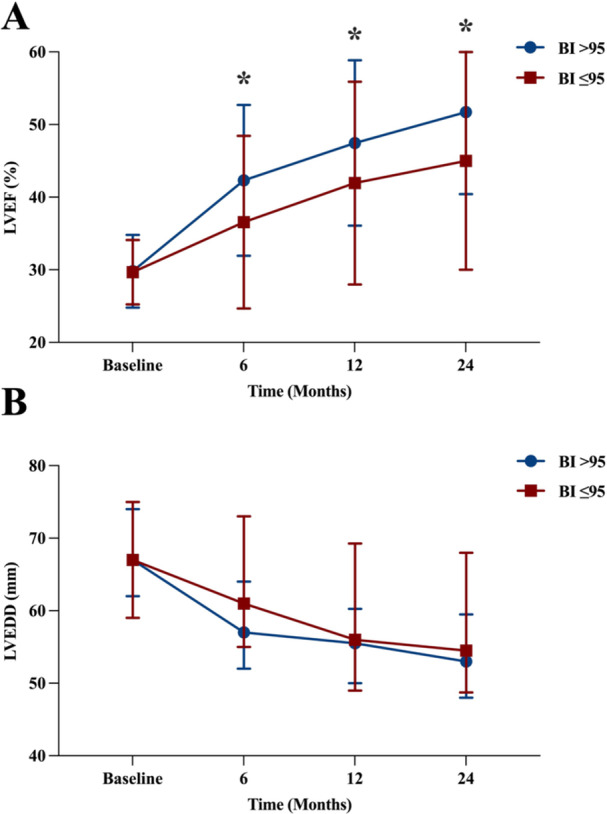
Comparison of Long‐term echocardiographic outcomes of patients receiving CRT stratified by BI levels (> 95 vs. ≤ 95). Comparison of Long‐term echocardiographic outcomes of patients receiving cardiac resynchronization therapy response (CRT) stratified by Barthel index (BI) levels (> 95 vs. ≤ 95). (A) The change trend of left ventricular ejection fraction (LVEF) measurements during follow‐up. (B) The change trend of left ventricular end‐diastolic diameter (LVEDD) measurements during follow‐up. *p < 0.05.

For the long‐term echocardiographic outcomes of these patients (Table 4), regarding LVEF, there was no significant difference in baseline levels between the BI > 95 and BI ≤ 95 groups (29.81% ± 5.01% vs. 29.67% ± 4.43%, *p* = 0.876); while LVEF improved over time in both groups (Figure 5A), the improvement was more pronounced in the BI > 95 group, with significantly higher LVEF in this group at 6 months (42.32% ± 10.39% vs. 36.56% ± 11.88%, *p* = 0.004), 12 months (47.46% ± 11.39% vs. 41.94% ± 13.96%, *p* = 0.048), and 24 months (51.71% ± 11.30% vs. 45.00% ± 14.98%, *p* = 0.044). As for LVEDD, baseline values were comparable between the two groups (*p* = 0.519), and though LVEDD decreased in both groups during follow‐up, the marginal lower LVEDD in the BI > 95 group at 6 months did not persist in later visits (Figure 5B). Overall, CRT recipients with BI > 95 showed more sustained LVEF improvements over long‐term follow‐up compared to those with BI ≤ 95, while the transient LVEDD difference in the BI > 95 group did not last.

**Table 4:** Comparison of long‐term echocardiographic outcomes of patients receiving CRT stratified by BI levels (> 95 vs. ≤ 95).

Variables	Patients receiving CRT with BI > 95	Patients receiving CRT with BI ≤ 95	*p*
*LVEDD, mm*			
Baseline	66.00 (62.00, 76.00)	65.00 (57.00, 72.75)	0.519
6 months	56.00 (49.00, 61.25)	60.49 ± 12.70	0.276
12 months	55.00 (50.00, 61.00)	56.00 (49.00, 67.75)	0.541
24 months	53.00 (48.00, 59.00)	54.50 (49.25, 65.50)	0.487
*LVEF, %*			
Baseline	29.81 ± 5.01	29.67 ± 4.43	0.876
6 months	42.32 ± 10.39	36.56 ± 11.88	0.004
12 months	47.46 ± 11.39	41.94 ± 13.96	0.048
24 months	51.71 ± 11.30	45.00 ± 14.98	0.044

Abbreviations: CRT, cardiac resynchronization therapy; LVEDD, left ventricular end‐diastolic diameter; LVEF, left ventricular ejection fraction.

## 4 Discussion

This study systematically explored the predictive value of the BI, a simple and economical tool for assessing activities of daily living, for CRT response and long‐term clinical outcomes in non‐ischemic HF patients with LBBB. Its key findings reveal three insights: first, a higher BI independently predicts a favorable CRT response; second, patients with BI > 95 exhibit significantly improved echocardiographic recovery, reduced risk of composite endpoints of all‐cause mortality and HFH (primarily driven by fewer HFH), and more sustained LVEF improvement compared to those with BI ≤ 95; third, the combination of BI, LVEDD, and LBBAP achieves the highest predictive efficacy for CRT response (AUC = 0.78), while LBBAP combined with BI > 95 confers the most favorable long‐term prognosis. These results provide novel insights into optimizing patient selection and prognostic stratification for CRT.

### 4.1 BI as a Predictor of CRT Response and Outcomes

The core value of this study lies in validating a low‐cost, accessible clinical parameter (BI) as a robust predictor of CRT outcomes, addressing the long‐standing challenge of identifying CRT responders using non‐invasive and widely applicable tools. The BI evaluates patients' actual daily living performance via face‐to‐face interviews, encompassing 10 items including feeding, grooming, bathing, dressing, bowel/bladder control, toilet use, mobility, transfers, and stairs with three scoring scales: 2‐level (0/5 points) for 2 items (grooming, bathing), 3‐level (0/5/10 points) for 6 items (feeding, dressing, bowel/bladder control, toilet use, and stairs), and 4‐level (0/5/10/15 points) for 2 items (mobility, transfers) [17]. Total scores (0‐100) denote severe (0−60), moderate (61−90), slight (91−99) dependence, or independence (100). Completable at the bedside in 5−10 min without specialized equipment, BI exhibits high reproducibility, making it suitable for routine clinical application in both stroke and HF populations [17, 18].

This study revealed that higher BI (per 5‐point increment; OR = 1.37, 95% CI 1.12−1.67, *p* = 0.002), larger LVEDD (OR = 0.96, 95% CI 0.92−0.98, *p* = 0.005), and LBBAP versus BiVP (OR = 2.88, 95% CI 1.53−5.41, *p* = 0.001) were independent predictors for nonischemic HF with LBBB who received CRT. The optimal cutoff value of BI = 95, derived from the Youden index and adjusted for clinical practicality (BI scores are recorded in 5−10‐point intervals). The cutoff of > 95 was chosen to balance sensitivity and specificity, making it more practical for routine clinical application than the statistically optimal but clinically unfeasible 97.5 cutoff. This cutoff offers clear operational guidance: patients with BI > 95 may be prioritized for CRT due to their higher likelihood of response, while those with BI ≤ 95 may require pre‐procedural optimization of HF management like enhancing medication adherence to improve functional status before CRT implantation. In terms of long‐term echocardiographic outcomes, the sustained improvement in LVEF among patients with BI > 95 (42.32% at 6 months, 47.46% at 12 months, 51.71% at 24 months) supports a potential association between functional independence and favorable myocardial recovery after CRT. In contrast, the transient reduction in LVEDD observed in the BI > 95 group at 6 months (without persistence in later follow‐up) raises the possibility that BI may better reflect the potential for myocardial contractile function recovery rather than durable structural reversal. While BI indeed reflects overall functional status, it provides incremental predictive value independent of cardiac structural parameters such as LVEDD, capturing systemic health and functional reserve not reflected by echocardiographic measures alone. This distinction highlights that functional status (assessed by BI) and structural remodeling (assessed by LVEDD) capture different aspects of HF pathophysiology, justifying their combined use in predictive models.

### 4.2 LBBAP Versus BiVP in CRT Response and Outcomes

Subgroup analysis and multivariate regression results of this study consistently confirm that LBBAP outperforms BiVP in CRT response and long‐term clinical outcomes. LBBAP is an independent predictor of favorable CRT response (adjusted OR = 2.88, 95% CI: 1.53−5.41, *p* = 0.001), a finding consistent with the results of Wang et al. and Vijayaraman et al., who demonstrated that LBBAP yielded greater LVEF improvement than BiVP in HF patients with nonischemic cardiomyopathy and LBBB [5, 6]. Additionally, this study demonstrates that LBBAP is associated with more favorable clinical outcomes than BiVP in HF patients receiving CRT, which is consistent with previous research findings [6, 7]. Furthermore, through stratification by BI, this study further confirms that HF patients who receiving LBBAP with BI > 95 achieve the most optimal prognosis (HR = 0.101, 95% CI: 0.010−1.027, Log‐rank *p* = 0.014). The mechanism underlying this advantage is that LBBAP directly captures the LBB, thereby restoring physiological left ventricular electromechanical synchronization [7]. LBBAP achieves this via septal lead implantation targeting the LBB area, resulting in a paced QRS morphology close to normal sinus rhythm and eliminating LBBB‐related intraventricular conduction delay. This finding implies that for patients with preserved daily living, LBBAP may maximize the benefits of CRT by better correcting ventricular dyssynchrony, while BiVP remains a viable option for those with BI ≤ 95 but may require closer post‐procedural monitoring. For patients with BI > 95 (functional independence), standard CRT implantation is recommended, with LBBAP preferred when anatomically feasible given its favorable long‐term outcomes. For those with BI ≤ 95 (functional dependence), pre‐procedural optimization via intensified anti‐HF medication and cardiac rehabilitation is advised. These patients also require closer post‐procedure monitoring for HF exacerbation and CRT non‐response, alongside timely device adjustment and supplementary treatments when necessary.

## 5 Conclusions

The BI is a simple, cost‐effective predictor of CRT response and prognosis in non‐ischemic HF patients with LBBB, and higher BI independently predicts favorable CRT response. BI > 95 reduces all‐cause mortality and HFH risk and improves cardiac function.

## 6 Limitations

This study is limited by its single‐center observational design with CRT strategy assigned based on physician experience rather than randomization, potentially introducing selection bias and restricted external validity. All primary analyses were performed using an as‐treated approach rather than an intention‐to‐treat analysis. Small subgroup sample sizes and a median 33.94‐month follow‐up period limit ultra‐long‐term outcome assessment. In addition, BI relies on subjective bedside evaluation, with possible inter‐rater variability especially for scores near the 95‐point cutoff. And the study only included non‐ischemic HF patients with LVEF ≤ 35% and LBBB, restricting generalizability to other HF subgroups.

## 7 Clinical Perspectives

This study validates the BI as a simple, cost‐effective tool for optimizing CRT management in non‐ischemic HF patients with LBBB, with key clinical implications: 1. CRT Candidate Selection: BI independently predicts CRT response (per 5‐point increment; OR = 1.37, 95% CI: 1.12−1.67, *p* = 0.002). The optimal cutoff of BI = 95 guides prioritization—patients with BI > 95 have a 75.38% response rate (vs. 44.44% for BI ≤ 95, *p* < 0.001) and should be prioritized, while those with BI ≤ 95 may benefit from pre‐procedural functional optimization. 2. Enhanced Predictive Efficacy: Combining BI with LVEDD and LBBAP achieves the highest CRT response prediction (AUC = 0.78), outperforming individual predictors and offering a readily implementable multimodal assessment in routine practice. 3. Prognostic Stratification and Therapy Personalization: BI > 95 reduces the risk of all‐cause mortality and HFH by 88% (adjusted HR = 0.116, *p* < 0.001) with sustained LVEF improvement. LBBAP combined with BI > 95 confers the most favorable prognosis (HR = 0.101, 95% CI: 0.010−1.027, *p* = 0.014), supporting LBBAP as the preferred strategy for functionally independent patients.

While the single‐center design and subjective nature of BI assessment limit its generalizability and reliability, future efforts should focus on validating BI in diverse HF populations via multicenter trials and developing objective digital tools to reduce assessment variability.

## Conflicts of Interest

The authors declare no conflicts of interest.

## Supporting information


Supporting File

## Data Availability

Study data are available from the corresponding author upon reasonable request.
