# Supplementary material for: The Barthel Index in Predicting Response to Cardiac Resynchronization Therapy and Clinical Outcomes in Patients With Heart Failure
Source: Clin Cardiol. 2026 Jul 9;49(7):e70389. doi: 10.1002/clc.70389 (PMC13348736; doi:10.1002/clc.70389)
Supplement: Supplementary file 1 — Supporting File [file CLC-49-e70389-s001.docx]

Supplementary table 1 Comparison of baseline characteristics between CRT patients enrolled for 2-year follow-up (2019-2023) and recently enrolled patients (2023-2024)

| Variables | Total (n = 249) | 2019-2023 CRT patients planned 2-year follow-up (n = 181) | 2023-2024 recently enrolled CRT patients (n = 68) | *P* |
| --- | --- | --- | --- | --- |
|  |  |  |  |  |
| Age, y | 59.85 ± 9.83 | 60.05 ± 9.83 | 59.31 ± 9.89 | 0.597 |
| Male, n (%) | 153 (61.45) | 109 (60.22) | 44 (64.71) | 0.517 |
| Hight, cm | 168.00 (160.00, 173.00) | 168.00 (160.00, 173.00) | 167.97 ± 6.88 | 0.574 |
| ﻿Weight, kg | 69.00 (60.00, 78.00) | 69.32 ± 12.80 | 65.00 (60.00, 75.00) | 0.254 |
| SBP, mmHg | 120.00 (110.00, 130.00) | 121.00 (110.00, 130.00) | 119.34± 14.32 | 0.301 |
| DBP, mmHg | 70.00 (64.00, 76.00) | 70.00 (64.00, 76.00) | 71.71 ± 9.15 | 0.349 |
| Smoke, n (%) | 84 (33.73) | 62 (34.25) | 22 (32.35) | 0.777 |
| Drink, n (%) | 59 (23.69) | 45 (24.86) | 14 (20.59) | 0.480 |
| Hypertension, n (%) | 105 (42.17) | 72 (39.78) | 33 (48.53) | 0.213 |
| Diabetes mellitus, n (%) | 56 (22.49) | 41 (22.65) | 15 (22.06) | 0.920 |
| NYHA≤2, n (%) | 90 (36.14) | 66 (36.46) | 24 (35.29) | 0.864 |
| History of HF, y | 3.00 (1.00, 8.00) | 3.00 (1.00, 8.00) | 3.00 (1.00, 7.00) | 0.491 |
| History of unexplained syncope or malignant VAs, n (%) | 31 (12.45) | 24 (13.26) | 7 (10.29) | 0.528 |
| NT-proBNP, ng/ml | 898.00 (475.75, 2054.50) | 898.00 (519.25, 2097.50) | 951.00 (461.50, 1787.25) | 0.806 |
| Barthel index >95, n (%) | 195 (78.31) | 138 (76.24) | 57 (83.82) | 0.196 |
| Intrinsic QRS duration (ms) | 178.23 ± 18.83 | 178.56 ± 18.69 | 177.37 ± 19.32 | 0.658 |
| Echocardiography |  |  |  |  |
| LVEDD, mm | 67.00 (62.00, 75.00) | 67.00 (62.00, 75.00) | 68.96 ± 10.32 | 0.770 |
| LVEF, % | 29.67 ± 4.84 | 29.62 ± 4.89 | 29.81 ± 4.74 | 0.783 |
| ﻿Medication |  |  |  |  |
| ﻿Beta blockers | 248 (99.60) | 180 (99.45) | 68 (100.00) | 1.000 |
| ﻿ACEI, ARB, ARNI | 223 (89.56) | 163 (90.06) | 60 (88.24) | 0.676 |
| ﻿Spironolactone | 241 (96.79) | 174 (96.13) | 67 (98.53) | 0.581 |
| SDLT2i | 201 (80.72) | 143 (79.01) | 58 (85.29) | 0.262 |
| ﻿ Diuretic | 224 (89.96) | 166 (91.71) | 58 (85.29) | 0.133 |
| CRT strategy, n (%) |  |  |  | 0.596 |
| BiVP | 125 (50.20) | 89 (49.17) | 36 (52.94) |  |
| LBBAP | 124 (49.80) | 92 (50.83) | 32 (47.06) |  |

Values are presented as mean ± SD or median (IQR) for continuous variables and numbers and percentages for categorical variables. Abbreviation: CRT, cardiac resynchronization therapy; SBP, systolic blood pressure; DBP, diastolic blood pressure; HF, heart failure; VAs, ventricular arrythmias; LVEDD, left ventricular end-diastolic diameter; LVEF, left ventricular ejection fraction; ACEI, angiotensin-converting enzyme inhibitor; ARB, angiotensin Ⅱ receptor blocker; ARNI, angiotensin receptor-neprilysin inhibitor; SGLT2i, sodium-glucose cotransporter 2 inhibitor; LBBAP, left bundle branch area pacing; BiVP, biventricular pacing.

Supplementary table 2 Predictors of all-cause mortality and HFH by univariate and multivariate Cox regression analyses

| Variables | Univariate Cox Regression | |  | Multivariate Cox Regression | | |
| --- | --- | --- | --- | --- | --- | --- |
|  | HR (95%CI) | *P* | |  | HR (95%CI) | *P* |
| Age, y | 0.99 (0.95-1.04) | 0.736 | |  |  |  |
| Male | 2.24 (0.72-6.96) | 0.162 | |  |  |  |
| Hight, cm | 1.05 (0.98-1.12) | 0.186 | |  |  |  |
| Weight, Kg | 1.01 (0.97-1.05) | 0.574 | |  |  |  |
| SBP | 0.98 (0.95-1.00) | 0.085 | |  | 1.01 (0.98-1.04) | 0.687 |
| DBP | 1.02 (0.97-1.06) | 0.531 | |  |  |  |
| Smoke | 1.27 (0.46-3.51) | 0.641 | |  |  |  |
| Drink | 1.56 (0.54-4.51) | 0.409 | |  |  |  |
| Hypertension | 0.32 (0.09-1.13) | 0.076 | |  | 0.61 (0.15-2.39) | 0.476 |
| Diabetes mellitus | 1.00 (0.19-5.40) | 0.997 | |  |  |  |
| NYHA≤2 | 1.35 (0.28-2.32) | 0.478 | |  |  |  |
| History of HF, y | 1.04 (0.97-1.12) | 0.300 | |  |  |  |
| History of unexplained syncope or malignant VAs | 3.94 (1.43-10.84) | 0.008 | |  | 6.57 (1.80-23.91) | 0.004 |
| NT-proBNP | 1.28 (1.11-1.48) | 0.143 | |  |  |  |
| Barthel index>95 | 0.20 (0.08-0.55) | 0.002 | |  | 0.12 (0.03-0.40) | <0.001 |
| Intrinsic QRS duration, ms | 0.99 (0.96-1.02) | 0.553 | |  |  |  |
| ﻿Echocardiography |  |  | |  |  |  |
| LVEDD, mm | 1.08 (1.04-1.12) | <0.001 | |  | 1.06 (1.01-1.12) | 0.049 |
| LVEF, % | 0.90 (0.82-0.99) | 0.035 | |  | 0.95 (0.83-1.10) | 0.510 |
| ﻿Medication |  |  | |  |  |  |
| ﻿Beta blockers | / | 0.998 | |  |  |  |
| ﻿ACEI, ARB, ARNI | 0.24 (0.01-4.37) | 0.337 | |  |  |  |
| ﻿Spironolactone | / | 0.731 | |  |  |  |
| SDLT2i | 0.71 (0.26-1.95) | 0.504 | |  |  |  |
| ﻿ Diuretic | 1.27 (0.17-9.65) | 0.816 | |  |  |  |
| LBBAP vs. BiVP | 0.28 (0.09-0.86) | 0.027 | |  | 0.24 (0.06-0.95) | 0.042 |

Abbreviation: HFH, heart failure hospitalization; SBP, systolic blood pressure; DBP, diastolic blood pressure; HF, heart failure; VAs, ventricular arrythmias; LVEDD, left ventricular end-diastolic diameter; LVEF, left ventricular ejection fraction; ACEI, angiotensin-converting enzyme inhibitor; ARB, angiotensin Ⅱ receptor blocker; ARNI, angiotensin receptor-neprilysin inhibitor; SGLT2i, sodium-glucose cotransporter 2 inhibitor; LBBAP, left bundle branch area pacing; BiVP, biventricular pacing.

Supplementary table 3 Predictors of CRT response (absolute LVEF increase ≥10%) by univariate and multivariate logistic regression analyses

| Variables | Univariate Logistic Regression | |  | Multivariate Logistic Regression | | |
| --- | --- | --- | --- | --- | --- | --- |
|  | OR (95%CI) | *P* | |  | OR (95%CI) | *P* |
| Age, y | 1.00 (0.98-1.03) | 0.984 | |  |  |  |
| Male | 0.78 (0.47-1.30) | 0.342 | |  |  |  |
| Height, cm | 0.97 (0.94-1.01) | 0.115 | |  |  |  |
| Weight, Kg | 0.99 (0.97-1.01) | 0.464 | |  |  |  |
| SBP | 1.00 (0.98-1.01) | 0.580 | |  |  |  |
| DBP | 1.02 (0.99-1.04) | 0.216 | |  |  |  |
| Smoke | 0.74 (0.44-1.25) | 0.263 | |  |  |  |
| Drink | 0.81 (0.45-1.46) | 0.488 | |  |  |  |
| Hypertension | 1.49 (0.90-2.47) | 0.123 | |  |  |  |
| Diabetes mellitus | 1.48 (0.81-2.70) | 0.202 | |  |  |  |
| NYHA≤2 | 1.02 (0.61-1.71) | 0.944 | |  |  |  |
| History of HF, y | 0.94 (0.89-0.99) | 0.017 | |  | 0.96 (0.91-1.02) | 0.173 |
| History of unexplained syncope or malignant VAs | 0.75 (0.35-1.60) | 0.458 | |  |  |  |
| Barthel index (per 5-point increment) | 1.26 (1.08-1.46) | 0.034 | |  | 1.27 (1.15-1.38) | 0.022 |
| NT-proBNP | 1.48 (0.78-2.17) | 0.400 | |  |  |  |
| Intrinsic QRS duration, ms | 1.00 (0.98-1.01) | 0.643 | |  |  |  |
| ﻿Echocardiography |  |  | |  |  |  |
| LVEDD, mm | 0.94 (0.92-0.97) | <0.001 | |  | 0.95 (0.92-0.98) | 0.001 |
| LVEF, % | 1.02 (0.97-1.08) | 0.414 | |  |  |  |
| ﻿Medication |  |  | |  |  |  |
| ﻿Beta blockers | / | 0.987 | |  |  |  |
| ﻿ACEI, ARB, ARNI | 1.21 (0.45-3.23) | 0.711 | |  |  |  |
| ﻿Spironolactone | 1.06 (0.26-4.34) | 0.936 | |  |  |  |
| SGLT2i | 1.42 (0.78-2.58) | 0.251 | |  |  |  |
| ﻿ Diuretic | 2.01 (0.85-4.75) | 0.109 | |  |  |  |
| LBBAP vs. BiVP | 2.08 (1.25-3.45) | 0.005 | |  | 1.94 (1.16-2.80) | 0.039 |

Abbreviation: CRT, cardiac resynchronization therapy; OR, odds ratio; SBP, systolic blood pressure; DBP, diastolic blood pressure; HF, heart failure; VAs, ventricular arrhythmias; LVEDD, left ventricular end-diastolic diameter; LVEF, left ventricular ejection fraction; ACEI, angiotensin-converting enzyme inhibitor; ARB, angiotensin Ⅱ receptor blocker; ARNI, angiotensin receptor-neprilysin inhibitor; SGLT2i, sodium-glucose cotransporter 2 inhibitor; LBBAP, left bundle branch area pacing; BiVP, biventricular pacing

Supplementary table 4 Predictors of CRT response (absolute LVEF increase ≥15%) by univariate and multivariate logistic regression analyses

| Variables | Univariate Logistic Regression | |  | Multivariate Logistic Regression | | |
| --- | --- | --- | --- | --- | --- | --- |
|  | OR (95%CI) | *P* | |  | OR (95%CI) | *P* |
| Age, y | 1.01 (0.98-1.04) | 0.501 | |  |  |  |
| Male | 0.69 (0.41-1.16) | 0.165 | |  |  |  |
| Height, cm | 0.98 (0.95-1.01) | 0.241 | |  |  |  |
| Weight, Kg | 1.00 (0.98-1.02) | 0.993 | |  |  |  |
| SBP | 1.00 (0.98-1.02) | 0.921 | |  |  |  |
| DBP | 1.01 (0.99-1.04) | 0.309 | |  |  |  |
| Smoke | 0.73 (0.42-1.27) | 0.266 | |  |  |  |
| Drink | 0.60 (0.32-1.13) | 0.113 | |  |  |  |
| Hypertension | 1.82 (1.08-3.05) | 0.023 | |  | 1.35 (0.76-2.40) | 0.312 |
| Diabetes mellitus | 1.76 (0.96-3.20) | 0.066 | |  | 1.47 (0.75-2.87) | 0.625 |
| NYHA≤2 | 0.89 (0.53-1.51) | 0.670 | |  |  |  |
| History of HF, y | 0.95 (0.90-1.01) | 0.100 | |  | 0.98 (0.92-1.04) | 0.472 |
| History of unexplained syncope or malignant VAs | 0.83 (0.38-1.81) | 0.967 | |  |  |  |
| Barthel index (per 5-point increment) | 1.26 (1.02-1.56) | 0.034 | |  | 1.31 (1.14-1.60) | 0.039 |
| NT-proBNP | 1.31 (0.24-2.85) | 0.792 | |  |  |  |
| Intrinsic QRS duration, ms | 0.99 (0.98-1.01) | 0.445 | |  |  |  |
| ﻿Echocardiography |  |  | |  |  |  |
| LVEDD, mm | 0.95 (0.92-0.98) | <0.001 | |  | 0.96 (0.93-0.99) | 0.011 |
| LVEF, % | 1.02 (0.97-1.08) | 0.417 | |  |  |  |
| ﻿Medication |  |  | |  |  |  |
| ﻿Beta blockers | / | 0.987 | |  |  |  |
| ﻿ACEI, ARB, ARNI | 0.92 (0.34-2.51) | 0.847 | |  |  |  |
| ﻿Spironolactone | 0.64 (0.16-2.62) | 0.543 | |  |  |  |
| SGLT2i | 1.22 (0.66-2.26) | 0.527 | |  |  |  |
| ﻿ Diuretic | 2.21 (0.85-5.74) | 0.104 | |  |  |  |
| LBBAP vs. BiVP | 2.47 (1.47-4.17) | <0.001 | |  | 2.04 (1.17-3.55) | 0.012 |

Abbreviation: CRT, cardiac resynchronization therapy; OR, odds ratio; SBP, systolic blood pressure; DBP, diastolic blood pressure; HF, heart failure; VAs, ventricular arrhythmias; LVEDD, left ventricular end-diastolic diameter; LVEF, left ventricular ejection fraction; ACEI, angiotensin-converting enzyme inhibitor; ARB, angiotensin Ⅱ receptor blocker; ARNI, angiotensin receptor-neprilysin inhibitor; SGLT2i, sodium-glucose cotransporter 2 inhibitor; LBBAP, left bundle branch area pacing; BiVP, biventricular pacing

Supplementary table 5 Comparison of baseline characteristics between patients treated with LBBAP or BiVP

| Variables | BiVP (n = 125) | LBBP (n = 124) | *P* |
| --- | --- | --- | --- |
|  |  |  |  |
| Age, y | 58.99 ± 10.36 | 60.71 ± 9.22 | 0.168 |
| Male, n (%) | 90 (72.00) | 63 (50.81) | <0.001 |
| Height, cm | 170.00 (165.00, 173.00) | 166.25 ± 8.25 | 0.064 |
| ﻿Weight, kg | 67.00 (60.00, 78.00) | 68.15 ± 12.06 | 0.413 |
| SBP, mmHg | 120.80 ± 16.41 | 120.41 ± 16.02 | 0.931 |
| DBP, mmHg | 71.27 ± 9.27 | 70.21 ± 10.10 | 0.390 |
| Smoke, n (%) | 50 (40.00) | 34 (27.42) | 0.036 |
| Drink, n (%) | 37 (29.60) | 22 (17.74) | 0.028 |
| Hypertension, n (%) | 51 (40.80) | 54 (43.55) | 0.661 |
| Diabetes mellitus, n (%) | 24 (19.20) | 32 (25.81) | 0.212 |
| NYHA≤2, n (%) | 44 (35.20) | 46 (37.10) | 0.755 |
| History of HF, y | 3.00 (1.00, 9.00) | 3.00 (1.00, 6.00) | 0.158 |
| History of unexplained syncope or malignant VAs, n (%) | 16 (12.80) | 15 (12.10) | 0.876 |
| NT-proBNP, ng/ml | 900.00 (547.00, 2108.00) | 896.00 (474.50, 1958.50) | 0.790 |
| Barthel index >95, n (%) | 102 (81.60) | 93 (75.00) | 0.206 |
| Intrinsic QRS duration (ms) | 179.82 ± 19.71 | 176.63 ± 17.84 | 0.181 |
| Echocardiography |  |  |  |
| LVEDD, mm | 70.00 (64.00, 77.00) | 66.08 ± 9.07 | <0.001 |
| LVEF, % | 29.14 ± 5.06 | 30.21 ± 4.56 | 0.080 |
| ﻿Medication |  |  |  |
| ﻿Beta blockers | 125 (100.00) | 123 (99.19) | 0.498 |
| ﻿ACEI, ARB, ARNI | 120 (96.00) | 112 (90.32) | 0.076 |
| ﻿Spironolactone | 121 (96.80) | 120 (96.77) | 1.000 |
| SGLT2i | 98 (78.40) | 95 (76.61) | 0.763 |
| ﻿ Diuretic | 118 (94.40) | 106 (85.48) | 0.019 |

Values are presented as mean ± SD or median (IQR) for continuous variables and numbers and percentages for categorical variables. Abbreviation: CRT, cardiac resynchronization therapy; SBP, systolic blood pressure; DBP, diastolic blood pressure; HF, heart failure; VAs, ventricular arrythmias; LVEDD, left ventricular end-diastolic diameter; LVEF, left ventricular ejection fraction; ACEI, angiotensin-converting enzyme inhibitor; ARB, angiotensin Ⅱ receptor blocker; ARNI, angiotensin receptor-neprilysin inhibitor; SGLT2i, sodium-glucose cotransporter 2 inhibitor; LBBAP, left bundle branch area pacing; BiVP, biventricular pacing.

Supplementary table 6 Predictors of response to LBBAP by univariate and multivariate logistic regression analyses

| Variables | Univariate Logistic Regression | |  | Multivariate Logistic Regression | | |
| --- | --- | --- | --- | --- | --- | --- |
|  | OR (95%CI) | *P* | |  | OR (95%CI) | *P* |
| Age, y | 1.01 (0.96-1.06) | 0.719 | |  |  |  |
| Male | 1.16 (0.47-2.87) | 0.752 | |  |  |  |
| Height, cm | 1.01 (0.95-1.06) | 0.849 | |  |  |  |
| Weight, Kg | 1.02 (0.98-1.06) | 0.339 | |  |  |  |
| SBP | 0.98 (0.96-1.01) | 0.252 | |  |  |  |
| DBP | 1.00 (0.96-1.05) | 0.892 | |  |  |  |
| Smoke | 0.83 (0.31-2.25) | 0.720 | |  |  |  |
| Drink | 0.53 (0.18-1.56) | 0.251 | |  |  |  |
| Hypertension | 1.99 (0.75-5.25) | 0.165 | |  |  |  |
| Diabetes mellitus | 1.31 (0.44-3.88) | 0.622 | |  |  |  |
| NYHA≤2 | 0.70 (0.26-1.84) | 0.465 | |  |  |  |
| History of HF, y | 0.92 (0.85-1.00) | 0.051 | |  | 0.91 (0.83-0.99) | 0.038 |
| History of unexplained syncope or malignant VAs | 0.40 (0.12-1.30) | 0.162 | |  |  |  |
| Barthel index (per 5-point increment) | 1.68 (1.15-2.45) | 0.007 | |  | 1.57 (1.13-2.17) | 0.007 |
| NT-proBNP | 1.02 (0.89-1.15) | 0.807 | |  |  |  |
| Intrinsic QRS duration, ms | 0.98 (0.96-1.01) | 0.130 | |  |  |  |
| ﻿Echocardiography |  |  | |  |  |  |
| LVEDD, mm | 0.96 (0.92-1.01) | 0.162 | |  |  |  |
| LVEF, % | 1.04 (0.94-1.14) | 0.483 | |  |  |  |
| ﻿Medication |  |  | |  |  |  |
| ﻿Beta blockers | / | 0.992 | |  |  |  |
| ﻿ACEI, ARB, ARNI | 0.37 (0.05-3.03) | 0.365 | |  |  |  |
| ﻿Spironolactone | / | 0.990 | |  |  |  |
| SGLT2i | 1.20 (0.42-3.39) | 0.735 | |  |  |  |
| ﻿ Diuretic | 2.62 (0.86-7.93) | 0.089 | |  | 3.22 (0.96-10.87) | 0.059 |

Abbreviation: OR, odds ratio; SBP, systolic blood pressure; DBP, diastolic blood pressure; HF, heart failure; VAs, ventricular arrhythmias; LVEDD, left ventricular end-diastolic diameter; LVEF, left ventricular ejection fraction; ACEI, angiotensin-converting enzyme inhibitor; ARB, angiotensin Ⅱ receptor blocker; ARNI, angiotensin receptor-neprilysin inhibitor; SGLT2i, sodium-glucose cotransporter 2 inhibitor; BiVP, biventricular pacing.

Supplementary table 7 Predictors of response to BiVP by univariate and multivariate logistic regression analyses

| Variables | Univariate Logistic Regression | |  | Multivariate Logistic Regression | | |
| --- | --- | --- | --- | --- | --- | --- |
|  | OR (95%CI) | *P* | |  | OR (95%CI) | *P* |
| Age, y | 1.00 (0.97-1.04) | 0.798 | |  |  |  |
| Male | 1.10 (0.50-2.41) | 0.810 | |  |  |  |
| Height, cm | 0.97 (0.92-1.03) | 0.295 | |  |  |  |
| Weight, Kg | 0.97 (0.95-1.00) | 0.083 | |  | 0.98 (0.95-1.01) | 0.177 |
| SBP | 1.01 (0.99-1.04) | 0.232 | |  |  |  |
| DBP | 1.02 (0.98-1.06) | 0.313 | |  |  |  |
| Smoke | 0.67 (0.32-1.37) | 0.271 | |  |  |  |
| Drink | 0.77 (0.35-1.66) | 0.498 | |  |  |  |
| Hypertension | 1.60 (0.77-3.30) | 0.208 | |  |  |  |
| Diabetes mellitus | 1.39 (0.56-3.48) | 0.477 | |  |  |  |
| NYHA≤2 | 1.10 (0.52-2.29) | 0.809 | |  |  |  |
| History of HF, y | 0.93 (0.86-1.00) | 0.064 | |  | 0.95 (0.88-1.04) | 0.251 |
| History of unexplained syncope or malignant VAs | 0.57 (0.20-1.64) | 0.295 | |  |  |  |
| Barthel index (per 5-point increment) | 1.24 (1.09-1.44) | 0.046 | |  | 1.26 (1.09-1.42) | 0.037 |
| NT-proBNP | 1.06 (0.91-1.17) | 0.545 | |  |  |  |
| Intrinsic QRS duration, ms | 0.99 (0.98-1.01) | 0.566 | |  |  |  |
| ﻿Echocardiography |  |  | |  |  |  |
| LVEDD, mm | 0.93 (0.90-0.97) | <0.001 | |  | 0.94 (0.90-0.98) | 0.006 |
| LVEF, % | 1.00 (0.94-1.08) | 0.929 | |  |  |  |
| ﻿Medication |  |  | |  |  |  |
| ﻿Beta blockers | / | 0.974 | |  |  |  |
| ﻿ACEI, ARB, ARNI | / | 0.987 | |  |  |  |
| ﻿Spironolactone | 1.28 (0.17-9.41) | 0.806 | |  |  |  |
| SGLT2i | 2.20 (0.92-5.24) | 0.075 | |  | 2.46 (0.93-6.53) | 0.071 |
| ﻿ Diuretic | 0.95 (0.20-4.44) | 0.950 | |  |  |  |

Abbreviation: OR, odds ratio; SBP, systolic blood pressure; DBP, diastolic blood pressure; HF, heart failure; VAs, ventricular arrhythmias; LVEDD, left ventricular end-diastolic diameter; LVEF, left ventricular ejection fraction; ACEI, angiotensin-converting enzyme inhibitor; ARB, angiotensin Ⅱ receptor blocker; ARNI, angiotensin receptor-neprilysin inhibitor; SGLT2i, sodium-glucose cotransporter 2 inhibitor; LBBAP, left bundle branch area pacing.
